# Establishing the role of the gut microbiota in susceptibility to recurrent urinary tract infections

**DOI:** 10.1172/JCI158497

**Published:** 2022-03-01

**Authors:** Colin J. Worby, Benjamin S. Olson, Karen W. Dodson, Ashlee M. Earl, Scott J. Hultgren

**Affiliations:** 1 Broad Institute of MIT and Harvard, Infectious Disease and Microbiome Program, Cambridge, Massachusetts, USA.; 2 Department of Molecular Microbiology and; 3 Center for Women’s Infectious Disease Research (cWIDR), Washington University School of Medicine, St. Louis, Missouri, USA.

## Introduction

The widespread use of antibiotics, in both healthcare and agriculture, has led to the emergence of antibiotic-resistant bacteria, decreasing our ability to effectively treat common infections. With predictions of antibiotic resistance reaching a tipping point, it is imperative that we develop novel, antibiotic-sparing medicines to avoid a future of increasing mortality due to currently treatable common infections. In the United States, 15% of antibiotics are prescribed for the treatment of urinary tract infections (UTIs) (1) affecting millions of women annually. For those suffering acute UTI, 25% experience recurrent UTIs (rUTIs) (1), involving several infections per year, that require multiple antibiotic courses. Recent history of a UTI is a known risk factor for rUTI (2), yet the mechanisms leading to recurrence are mostly unknown. In addition, antibiotic resistance is making UTIs harder to treat and often necessitates using broad-spectrum antibiotics. Ironically, antibiotic use is also a significant risk factor for a UTI (3), possibly due to associated deleterious effects on the gut microbiota, among which most uropathogens reside. That approximately 50% of rUTIs are caused by the same strain that caused the initial infection (4) argues for a host-associated reservoir that is not adequately cleared by current treatments. Thus, there is an urgent need to better understand uropathogen dynamics within host-associated reservoirs to develop treatment options that limit morbidity and antibiotic consumption.

UTIs are most commonly caused by uropathogenic *Escherichia coli* (UPEC), which reside in the gut and can ascend the urinary tract to cause infection. This process is driven by physical manipulation, such as sexual intercourse, which is a clear risk factor for UTI (2, 5). Studies have shown that the majority of UTI-causing UPEC are resident in the gut at the time of UTI (6) and are often the dominant *E*. *coli* strain in the gut. Many studies have explored the role of host (behavior, ref. 2; genetics, ref. 7) and pathogen (genotype/phenotype, ref. 8), and it appears likely that an integration of both defines rUTI risk. However, despite the established role of the gut as a UPEC reservoir, we are only beginning to understand UPEC-gut-microbiota interactions and how these interactions may modulate rUTI susceptibility.

**Figure 1:**
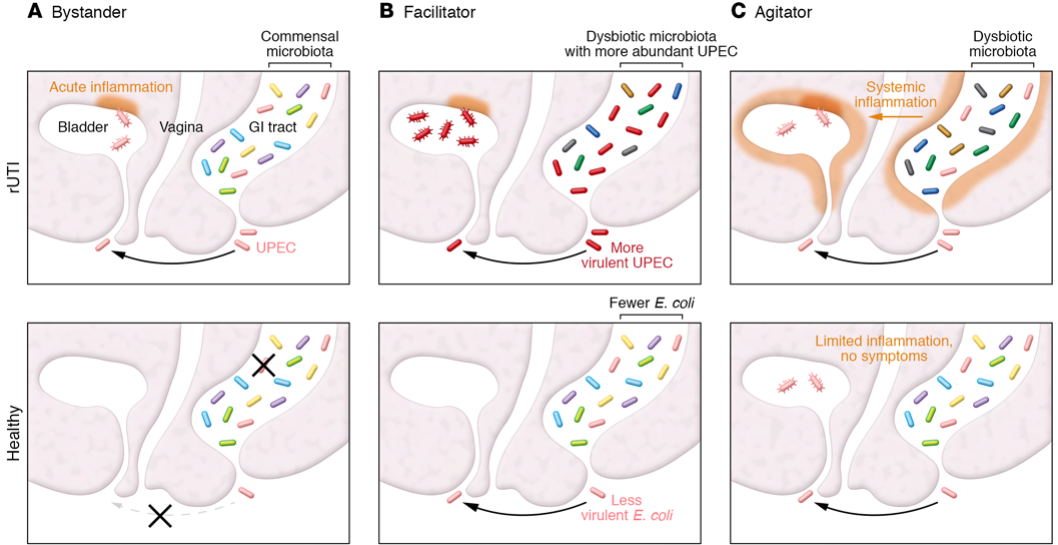
The role of the gut microbiome in rUTI. (**A**) Bystander: gut does not affect susceptibility to rUTI. Other mechanisms prevent either UPEC colonization of the gut or invasion of the bladder in healthy women. (**B**) Facilitator: dysbiotic gut facilitates UPEC colonization in rUTI. More abundant and/or more urovirulent *E*. *coli* (darker shade of red) in the guts of rUTI women increase the risk of bladder infection. (**C**) Agitator: UPEC invasion of the bladder occurs in all women, but interactions between a dysbiotic gut and the host immune system result in increased inflammation and symptom severity in rUTI women. The bottom panel illustrates some of the many states of the gut-bladder axis in otherwise healthy people. Hair-like fibers are type 1 pili. GI, gastrointestinal.

Here, we consider three hypotheses regarding the role of the gut: (a) the gut microbiota does not directly affect UTI risk, serving only as a passive reservoir for UPEC (gut as bystander; Figure 1A); and (b) the gut microbiota provides a differentially hospitable environment for UPEC, thus modulating the risk of gut colonization and subsequent successful colonization of the bladder (gut as facilitator; Figure 1B); and/or (c) host-microbiota interactions in the gut affect the systemic immune system to cause differential response to bacterial invasion of the bladder (gut as agitator; Figure 1C).

## The gut as a bystander

If the gut is merely a passive reservoir that UPEC may transiently inhabit but not influence, we would anticipate broadly similar microbiome composition profiles between healthy and rUTI women. While current evidence remains limited due to the lack of appropriate cohort studies, recent work has shown that children with UTIs (9) and kidney transplant patients with bacteriuria (10) have differential microbiome structures compared with respective control cohorts, suggesting a link between the resident microbiota and uropathogen gut colonization and/or transmission to the bladder. Furthermore, our recent longitudinal cohort study identified reduced microbial diversity and lower levels of butyrate-producing bacteria in the guts of women with rUTI history compared with healthy controls (11). Antibiotic treatment of UTIs is certainly a confounder in such studies due to the resulting perturbations of the gut microbiota associated with repeated exposure. As such, differences in composition may reflect the impact of UTI treatment rather than a signal of heightened susceptibility. Nevertheless, it has been shown that perturbation of the microbiota may affect UTI occurrence; fecal microbiota transplants (FMTs) for *Clostridium* *difficile* infection had the collateral effect of decreasing UTI frequency in women with a history of rUTI (12, 13). While the mechanism of rUTI risk reduction remains unclear, this work highlights that the gut is unlikely to function solely as a bystander in rUTI susceptibility.

## The gut as a facilitator

The concept of “colonization resistance” has gained traction in recent years, with the notion that a perturbed or dysbiotic gut may be more permissive to pathogen colonization when compared to the “resistance” provided by a healthy, diverse microbiota (14, 15). In a murine model for UPEC gut colonization, oral streptomycin treatment is required for UPEC to effectively colonize the gut (16), hypothetically, due to loss in colonization resistance from the commensal microbiota. Similarly, depletion of commensal microbiota, and, in particular, butyrate-producing bacteria, can lead to increased levels of *Salmonella* and *C*. *difficile* in mice and humans, respectively (17, 18). Diminished gut colonization resistance to UPEC would provide increased opportunity to invade the bladder and cause infection. Indeed, uropathogen gut abundance is a risk factor for UTI in kidney transplant patients (19), while an intestinal *E*. *coli* “bloom” may precede many infections (20). Determining whether these blooms are due to transient changes in the microbiota leading to diminished colonization resistance or to other changes in the gut requires further investigation. In addition to colonization resistance, the gut may influence rUTI susceptibility through modulation of the transcriptional activity of resident UPEC. In vitro studies have shown that differing levels of short chain fatty acids (SCFAs) can regulate expression of enterohemorrhagic *E*. *coli* virulence factors as well as pathosymbiont *E*. *coli* associated with inflammatory bowel disease (21). These include known virulence factors for UPEC adhesion to, and invasion of, host cells (FimH) and motility (FliC). While much work is still required to explore the causal links among rUTI, UPEC colonization resistance, and the gut microbiome, evidence is mounting that perturbations of the gut microbiota modulate the quantity and the virulence of UPEC.

## The gut as an agitator

A range of clinical disorders are now recognized to be driven, at least in part, by the gut microbiome, and interactions between the gut and distal organs are becoming increasingly well characterized. Metabolites produced in the gut can affect distal organs; for instance, recent work suggests that loss of SCFA-producing bacteria from the gut leads to inflammatory airway conditions (22), which are ameliorated by oral SCFA supplementation (23). Similarly, rheumatoid arthritis has been associated with depleted levels of butyrate producers (24, 25). Various commensal and pathogenic bacteria also appear to directly affect immune system programming through interactions at the gut epithelium. *Helicobacter pylori,* for instance, has been shown to decrease murine allergic airway disease by directly activating regulatory T cells (26).

Given these dynamics, we question whether the “gut-bladder axis” comprises not only the well-characterized direct transit of uropathogen from gut to bladder, but also indirect interactions via the systemic immune system. Distal inflammation at the bladder may be affected by SCFAs, or other metabolites, produced in the gut, resulting in increased bladder or uroepithelium inflammation upon infection. Whether UPEC gut presence alters the immune system’s response to eventual bladder exposure remains an open question. Our recent work revealed a significant depletion of butyrate producers in women with rUTI history regardless of immediate UTI status as well as tentative evidence for differential immune markers at healthy time points compared with a healthy cohort (11). While considerable work is needed to further explore these dynamics, we propose that the gut-bladder axis may be an as yet overlooked, but relevant, driver of rUTI susceptibility.

## Implications for treatment

Evidence that the microbiome plays a role in rUTI susceptibility is mounting. While the exact mechanisms are unestablished and likely complex, this opens up new targets for treatment and prophylaxis. The FMT-associated reductions in rUTIs among *C*. *difficile* patients is an encouraging sign that microbiome therapeutics could be successful. Future FMT trials focused on otherwise healthy rUTI women would clarify whether untargeted microbiome therapies could be beneficial. Concurrently, the gut-bladder axis highlights a further limitation of existing antibiotic treatments. Not only is antibiotic treatment a known risk factor for rUTI, antibiotics can cause significant and long-lasting perturbations of the gut microbiome (27, 28). If indeed UTI antibiotics generate, or maintain, a state of gut dysbiosis, this represents a vicious circle of treatment enhancing susceptibility to future infection. Small-molecule therapeutics may offer an opportunity to break this cycle by targeting only the UPEC subpopulation in the gut (29). While continued treatment may be required due to reexposure to UPEC from external sources, abstinence from antibiotics may allow the microbiome to return to a healthy state. Thus, targeting a human reservoir represents a promising new research avenue.

## Future research

As noted throughout, there is a clear lack of data regarding interactions among the gut microbiota, gut resident UPEC, and distal effects on the bladder. Considerable efforts will be required to untangle this complex system. However, there are also fundamental knowledge gaps regarding UPEC dynamics that would help to elucidate the role of the gut. It is known that women without rUTI history can carry UPEC-like strains in the gut. Do UPEC-like strains carried by healthy women differ genomically or transcriptionally from those causing infection? Any differences could point to the gut facilitating distinct phenotypic populations. How frequently do such strains transfer to the bladder without causing reportable symptoms? If this is common in healthy women, differential immune response is a compelling explanation for divergent symptomatic outcomes. Comprehensive genomic and transcriptomic surveillance of fecal and urine strains in rUTI patients and control women could offer significant insights into UTI pathogenesis and pathology. The gut microbiome almost certainly acts as a facilitator and/or agitator driving rUTI. A more complete understanding of these mechanisms is essential for the development of novel antibiotic-sparing treatments and prophylaxis.

## Version 1 03/01/2022

Electronic publication
